# Effects of a Web-Based Personalized Intervention on Physical Activity in European Adults: A Randomized Controlled Trial

**DOI:** 10.2196/jmir.4660

**Published:** 2015-10-14

**Authors:** Cyril FM Marsaux, Carlos Celis-Morales, Rosalind Fallaize, Anna L Macready, Silvia Kolossa, Clara Woolhead, Clare B O'Donovan, Hannah Forster, Santiago Navas-Carretero, Rodrigo San-Cristobal, Christina-Paulina Lambrinou, George Moschonis, Agnieszka Surwillo, Magdalena Godlewska, Annelies Goris, Jettie Hoonhout, Christian A Drevon, Yannis Manios, Iwona Traczyk, Marianne C Walsh, Eileen R Gibney, Lorraine Brennan, J Alfredo Martinez, Julie A Lovegrove, Michael J Gibney, Hannelore Daniel, John C Mathers, Wim HM Saris

**Affiliations:** ^1^ Department of Human Biology School of Nutrition and Translational Research in Metabolism (NUTRIM) Maastricht University Medical Centre + (MUMC+) Maastricht Netherlands; ^2^ Human Nutrition Research Centre Institute of Cellular Medicine Newcastle University Newcastle Upon Tyne United Kingdom; ^3^ Hugh Sinclair Unit of Human Nutrition and Institute for Cardiovascular and Metabolic Research University of Reading Reading United Kingdom; ^4^ Zentralinstitut für Ernährungs- und Lebensmittelforschung (ZIEL) Research Center of Nutrition and Food Sciences Biochemistry Unit Technische Universität München München Germany; ^5^ University College Dublin (UCD) Institute of Food and Health University College Dublin Belfield Dublin Ireland; ^6^ Department of Nutrition, Food Science and Physiology Centre for Nutrition Research University of Navarra Pamplona Spain; ^7^ Centro de Investigación Biomédica en Red-Fisiopatología de la Obesidad y Nutrición (CIBERobn) Instituto de Salud Carlos III Madrid Spain; ^8^ Department of Nutrition and Dietetics Harokopio University Athens Greece; ^9^ National Food & Nutrition Institute (IZZ) Warsaw Poland; ^10^ Personal Health Solutions Philips Consumer Lifestyle Amsterdam Netherlands; ^11^ Experiences Research Department Philips Research Eindhoven Netherlands; ^12^ Department of Nutrition, Institute of Basic Medical Sciences Faculty of Medicine University of Oslo Oslo Norway

**Keywords:** physical activity, eHealth, randomized controlled trial, personalized nutrition, genotype, phenotype, Internet

## Abstract

**Background:**

The high prevalence of physical inactivity worldwide calls for innovative and more effective ways to promote physical activity (PA). There are limited objective data on the effectiveness of Web-based personalized feedback on increasing PA in adults.

**Objective:**

It is hypothesized that providing personalized advice based on PA measured objectively alongside diet, phenotype, or genotype information would lead to larger and more sustained changes in PA, compared with nonpersonalized advice.

**Methods:**

A total of 1607 adults in seven European countries were randomized to either a control group (nonpersonalized advice, Level 0, L0) or to one of three personalized groups receiving personalized advice via the Internet based on current PA plus diet (Level 1, L1), PA plus diet and phenotype (Level 2, L2), or PA plus diet, phenotype, and genotype (Level 3, L3). PA was measured for 6 months using triaxial accelerometers, and self-reported using the Baecke questionnaire. Outcomes were objective and self-reported PA after 3 and 6 months.

**Results:**

While 1270 participants (85.81% of 1480 actual starters) completed the 6-month trial, 1233 (83.31%) self-reported PA at both baseline and month 6, but only 730 (49.32%) had sufficient objective PA data at both time points. For the total cohort after 6 months, a greater improvement in self-reported total PA (*P*=.02) and PA during leisure (nonsport) (*P*=.03) was observed in personalized groups compared with the control group. For individuals advised to increase PA, we also observed greater improvements in those two self-reported indices (*P*=.006 and *P*=.008, respectively) with increased personalization of the advice (L2 and L3 vs L1). However, there were no significant differences in accelerometer results between personalized and control groups, and no significant effect of adding phenotypic or genotypic information to the tailored feedback at month 3 or 6. After 6 months, there were small but significant improvements in the objectively measured physical activity level (*P*<.05), moderate PA (*P*<.01), and sedentary time (*P*<.001) for individuals advised to increase PA, but these changes were similar across all groups.

**Conclusions:**

Different levels of personalization produced similar small changes in objective PA. We found no evidence that personalized advice is more effective than conventional “one size fits all” guidelines to promote changes in PA in our Web-based intervention when PA was measured objectively. Based on self-reports, PA increased to a greater extent with more personalized advice. Thus, it is crucial to measure PA objectively in any PA intervention study.

**Trial Registration:**

ClinicalTrials.gov NCT01530139; http://clinicaltrials.gov/show/NCT01530139 (Archived by WebCite at: http://www.webcitation.org/6XII1QwHz)

## Introduction

Physical inactivity is one of the major risk factors for noncommunicable diseases [1]. It has been estimated that in 2008, approximately 7.3% of the 9.2 million deaths occurring in Europe were attributed to physical inactivity compared with 3.7% attributed to obesity [2]. Increasing physical activity (PA) continues to be a public health priority. Although public knowledge of the health benefits of regular PA is good and the recommendation of “30 min per day of activity most days of the week” is recognized widely, recent data from the World Health Organization (WHO) suggest that 35% of European adults do not meet PA recommendations [3].

Finding effective ways to increase PA is challenging. The limited success of "one size fits all" PA promotion programs may be partly due to the fact that inactive individuals are unaware that their current PA is inadequate [4,5]. Thus, providing personalized PA feedback may be more effective in increasing PA than a nonpersonalized conventional approach. Internet-based interventions for PA may have potential to increase levels of PA because large numbers of physically inactive individuals can be reached. However, it has been pointed out that positive effects were quite small, and might not be sustained in the long term. Furthermore, objective PA measurements and greater sample sizes are required [6-9].

Although many studies have used self-reports, such as the Baecke questionnaire or the International Physical Activity Questionnaire (IPAQ) [10], objective measurements of PA are more reliable [11]. Developments of PA measurement devices in the last decade have improved quantification of PA in free-living subjects. Accelerometers, for instance, have become popular because they can be worn without major inconvenience, require little effort by the user, and are compatible with most daily activities. Because they can record data for up to several weeks and measure PA accurately [12,13], they are useful research tools. In spite of this, few studies comparing tailored PA advice with nontailored or no advice have included these objective measures of PA [14-17].

Whether personalized PA feedback promotes behavioral change remains unclear. We used data collected during the Food4Me Study, which was registered at ClinicalTrials.gov (NCT01530139), to investigate the impact of different levels of personalization on PA change, using phenotypic and genotypic information to tailor the PA advice (see also Multimedia Appendix 1). We hypothesized that individually tailored advice would lead to greater and more sustained changes in PA, and that the intervention would be more effective as the level of personalization increased.

## Methods

### Study Design

Full details of the study protocols have been described elsewhere [18]. Briefly, the Food4Me proof-of-principle study was a 6-month, 4-arm, randomized controlled trial (RCT) conducted across seven European countries to compare the effects of three levels of personalized advice with standard population advice on health-related outcomes. The intervention was designed to emulate an Internet-based service [19] and aimed to answer the following primary questions: (1) does personalization of dietary and PA advice result in bigger improvement in diet and PA compared with nonpersonalized, conventional guidelines? And (2) is personalization based on individualized phenotypic or genotypic information more effective in assisting and/or motivating participants to make and sustain appropriate healthy changes than personalization based on analysis of baseline diet and PA alone? To answer these questions, participants were randomized to a control group (Level 0) or to one of three personalized intervention groups with increasingly more detailed personalized advice (Levels 1 to 3) for 6 months. The levels are described in Textbox 1.

**Description of control and intervention groups and their levels of personalization in the Food4Me Study.**

Levels of personalization

- Level 0 (L0; control group): nonpersonalized advice based on (European) general guidelines for diet and PA
- Level 1 (L1): personalized advice based on individual dietary intake and PA alone
- Level 2 (L2): personalized advice based on individual dietary intake, PA, and phenotypic data
- Level 3 (L3): personalized advice based on individual dietary intake, PA, and phenotypic and genotypic data

In the personalized groups, personalization of the PA advice was greater in L2 and L3 compared with L1: it was linked to phenotypic data (waist circumference, blood total cholesterol; L2 and L3) and genotypic data (fat mass- and obesity-associated gene, *FTO*; L3). See also the section Physical Activity Feedback below.

### Outcomes

We focus here on PA after 3 and 6 months of intervention. PA is presented using both objective (accelerometer) and self-reported (PA questionnaire) data. Other outcomes included dietary intake, but are not within the scope of this paper.

### Recruitment

We aimed to recruit 1540 participants aged ≥18 years in seven European countries—Germany, Greece, Ireland, the Netherlands, Poland, Spain, and the United Kingdom [18]. Subjects were ineligible to take part in the study if they had no or limited access to the Internet, were following a prescribed diet, or had altered nutritional requirements because of a medical condition. The ethics committee from each recruiting center approved the study protocol. Between August 2012 and August 2013, 1607 adults (653 men, 40.63%; and 954 women, 59.37%) were randomized to the intervention. All participants gave informed consent digitally before participating in the study.

### Measures

Data were collected using standard operating procedures in all seven countries [18]. Participants received study kits by post, containing all necessary materials (including an accelerometer) to perform measurements at home, but used their own scales to measure body weight. Printed and digital instructions, as well as online videos, were available for all participants in the languages of all seven countries.

### Objectively Measured Physical Activity

#### Objective Physical Activity Monitoring

Habitual PA was assessed objectively using the TracmorD triaxial accelerometer (Philips Consumer Lifestyle, the Netherlands) [20,21]. The device is small (3.2 × 3.2 × 0.5 cm), light (12.5 g), waterproof to a depth of 30 m, has a battery life of 3 weeks, and can record data for up to 22 weeks.

Participants activated the TracmorD accelerometer by creating an account online, installing an app on their computer, and connecting the device to the computer using the USB adapter provided. Upon activation, men could choose between three wearing positions—pocket, belt, or necklace—and women between four wearing positions—pocket, belt, necklace, or bra. Participants could change their wearing position after informing the research team, who would update the position in the online system. Participants were instructed to wear the accelerometer every day during waking hours, except when taking a shower, for the entire duration of the study. Participants uploaded data every 2 weeks by connecting their monitor to their computer. Researchers checked this regularly and sent reminders to participants, if necessary. The data were transferred in real time and stored on a secured server.

#### Objective Physical Activity Data Processing

Data were recorded with a time-sampling interval of 1 minute. A day was considered valid if the participant had worn the TracmorD for at least 10 hours but not longer than 18 hours. Wear time was defined as 24 hours minus nonwear time. To define nonwear time, we adapted the recommendations of Choi et al [22] to the TracmorD. Physical activity level (PAL)—the ratio of total energy expenditure to basal metabolic rate—data per minute were estimated from activity counts [20]. Nonwear time was defined by an interval of at least 90 consecutive minutes of PAL per minute values below 1.3889, allowing for 2-minute intervals of values above the threshold, with the upstream or downstream 30 consecutive values below the threshold (for detection of artifactual movements). R software version 3.1.2 [23] was used for all data handling.

#### Objective Physical Activity Variables

Daily PAL, activity energy expenditure (AEE), and time spent in different PA intensities were derived from the accelerometer.

PAL-per-day calculations were based on the work by Bonomi et al [20]:

PAL = 1.354 + (256 × 10^-9^) × counts_day_ (1)

where counts_day_ are the sum of minute-by-minute activity counts over 24 hours. However, the wearing position is taken into account, using the belt position as a reference and applying a correction factor for the other positions.

AEE per day was calculated as follows:

AEE = (0.9 × PAL per day - 1) × BMR (2)

where the daily basal metabolic rate (BMR) is estimated using the Oxford equations developed by Henry, based on sex, age, and weight [24].

Classification into sedentary, and light-, moderate-, and vigorous-intensity PA (LPA, MPA, and VPA, respectively) was based on the application of thresholds for AEE: 0.025, 0.05, and 0.1 kcal/(kg×min) corresponding to 1.5, 3, and 6 metabolic equivalents (METs), respectively. Sedentary time and time spent in LPA, MPA, and VPA were determined by summing the time during which AEE per minute met the criterion for the appropriate intensity. Finally, moderate-equivalent PA was defined as follows:

Moderate-equivalent PA = MPA + 2 × VPA (3)

to account for the fact that 1 minute of VPA is equivalent to 2 minutes of MPA [25]. Moderate-equivalent PA duration data were also calculated for activity occurring in modified bouts of 10 minutes (ie, with allowance for interruptions of ≤2 minutes at a lower PA intensity) [26].

PA estimates at baseline, month 3, and month 6 were calculated over a 2-week period at each time point. This 2-week assessment period occurred before any feedback was given to participants. Sufficient PA data at each time point was defined as having at least 3 valid weekdays and 2 valid weekend days of accelerometer wear during the 2-week assessment period. For individuals with sufficient PA data, mean data per day were calculated for all objective PA variables using all valid week and weekend days of the assessment period as follows:

Mean = (mean for weekdays × 5 + mean for weekend days × 2) / 7 (4).

For sedentary time and time spent in LPA, MPA, VPA, and moderate-equivalent PA, weekly estimates were also calculated as follows:

Mean = (mean for weekdays × 5 + mean for weekend days × 2) (5).

### Self-Reported Physical Activity

At baseline, month 3, and month 6, participants completed the Baecke questionnaire online [27] based on their PA during the last month. The Baecke questionnaire is a short, validated questionnaire assessing habitual PA according to the context in which it occurs and is organized into three sections: (1) PA at work, (2) sport, and (3) during leisure time excluding sport [27-29]. Indices for these three PA categories—work index, sport index, and leisure time (nonsport) index, each ranging from 1 to 5—as well as a total activity index—sum of the three previous indices, ranging from 3 to 15—were calculated according to the questionnaire protocol [27].

### Anthropometrics

Participants self-measured their height, weight, and waist circumference, and uploaded their measurements directly onto their personal Food4Me online account [18]. Validation of self-reported sociodemographic and anthropometric measures have been described elsewhere [30].

### Genotyping

Participants collected a buccal cell sample at baseline, using Isohelix SK-1 DNA buccal swabs and Isohelix Dri-capsules (LGC Genomics, Hertfordshire, UK). Samples were returned to their recruiting center and shipped to LGC Genomics, who extracted the DNA and used competitive allele-specific polymerase chain reaction (KASP) genotyping assays to provide biallelic scoring of single nucleotide polymorphism (SNP) rs9939609 in the *FTO* gene.

### Physical Activity Feedback

All participants received a feedback report at months 0 and 3 via email in PDF. Reports were also available on the participant’s personal Food4Me account. Participants in personalized groups (L1, L2, and L3) received personalized feedback based on accelerometer data (or self-reported data if accelerometer data were not available), whereas controls (L0) received a PDF containing general guidelines (see the following section). At each time point, researchers calculated the average PAL based on 2 weeks of accelerometer data collection for each participant and used it in the derivation of the PA feedback for personalized groups (L1 to L3). The feedback report was sent 1 to 2 weeks after this 2-week measuring period. For L0, the same generalized advice was sent at months 0 and 3. After completing the study, all participants received a personalized report based on the 6-month intervention.

### Cutoffs Definition

PA was defined as adequate if objective PAL was ≥1.8. A value of PAL ≥1.5 and <1.8 was considered low and a PAL of <1.5 was considered very low. If accelerometer data were not available at the time of feedback, the total activity index of the Baecke questionnaire was used instead of PAL with the following cutoffs: ≥8.5 (adequate PA), ≥5.5 to <8.5 (low), and <5.5 (very low).

### Derivation of Feedback Messages in Relation to Physical Activity

#### Level 0 (Controls)

Participants in the control group received the nonpersonalized advice that they should be physically active at least 150 minutes per week.

Feedback reports in personalized groups contained specific messages, selected according to standardized algorithms, based on subjects’ characteristics and their allocated group.

#### Level 1

In the PA section of the personalized report, current level of PA was indicated with a mark on a three-color line, based on the cutoffs defined above: red area (very low PAL), amber (low PAL), and green (adequate PAL). The report included tailored advice to increase strongly, increase, or maintain PA based on current PAL and body mass index (BMI), as well as tips on how to be (more) physically active. Participants had access to additional information about PA and tips online on their personal Food4Me account. Hyperlinks to this section of the website were included in the tailored report and participants were encouraged to visit the webpage.

#### Level 2

Participants in L2 had access to the same information as those in L1. However, the specific PA message in the personalized report was based on current PAL and BMI as well as on individuals’ waist circumference and blood total cholesterol.

#### Level 3

Participants in L3 had access to the same information as those in L1 and L2, in addition to whether they carried the risk allele (A) for the *FTO* gene; this information was included in the specific PA message alongside current PAL, BMI, waist circumference, and blood total cholesterol. For example, an inactive obese L3 participant with *FTO* risk (AA or AT genotype), high waist circumference, and high total cholesterol would receive the following advice:

Your BMI is greater than the recommended healthy range (...). Your waist circumference is also higher than recommended (...). We recommend reducing your body weight and waist circumference to a healthy normal range because you have a genetic variation that can benefit by reducing these two obesity markers (...). Also, your physical activity level is too low; improving your physical activity level will help you to reduce your weight. Your fasting cholesterol level was above the recommended level and we advise you to go to the G.P. [general practitioner] to get this re-checked (...). Become more physically active; to maintain weight loss, 60-90 minutes of moderately intense aerobic activities, such as brisk walking, swimming or cycling, on most days of the week, is recommended. This will also help to lower cholesterol levels.

PA feedback for L3 participants not carrying the risk for *FTO* was similar to that of L2 participants. However, L3 participants all received information on both *FTO* and 4 other diet-related genetic variants, whereas L2 participants did not receive any genetic-based information. More details of the feedback reports and the Food4Me website are given in the supplementary material (see Multimedia Appendix 2) and elsewhere [18].

### Statistical Analysis

Data were analyzed on an intention-to-treat basis. We defined 3 orthogonal contrasts to answer our research questions: first comparing L0 with L1 to L3, then L1 with L2 and L3, and finally L2 with L3. More specifically, to answer the first research question—Is personalized advice more effective than the conventional one size fits all?—intervention effects on PA variables were assessed. We used robust multiple linear regression analysis, based on computation of SMDM estimates [31] to account for violation of the normality assumption, with baseline PA variable, sex, age, country, smoking, baseline BMI, baseline season, and change in body weight as covariates. For accelerometer-derived PA variables, change in accelerometer wear time was included as an additional covariate. The principal assessment of intervention used Contrast 1 comparing L0 with the mean of L1 to L3. First, a generic approach was used where intervention effects for the total cohort were investigated. Second, a targeted approach was used in which the intervention effects on PA, only for participants who received advice to increase their PA, were investigated. For the second part, outcomes for those who received tailored advice targeting PA were compared with the subset of matched L0 (control) participants (ie, controls who would have received personalized advice to increase PA if they had been in a personalized group instead of L0). These matched L0 participants were selected by applying the same algorithm used for individuals in personalized groups.

The second research question—"Is personalization based on individualized dietary, phenotypic, or genotypic information more effective in promoting changes in PA than personalization based on diet and PA alone?”—was tested using two other contrasts. For Contrast 2, comparison of L1 with L2 and L3 tested whether personalization based on phenotypic and/or genotypic information differed from that based on dietary and PA assessment only. For Contrast 3, comparison of L2 with L3 tested whether the addition of genotypic information promoted a greater increase in PA than when using phenotypic, dietary, and PA information only. The analyses outcomes were the same PA variables as for Contrast 1 and both generic and targeted approaches were used.

Sensitivity analyses were performed to compare dropouts with completers and noncompliant (ie, those with too few valid days) with compliant participants. These analyses were performed using robust multiple linear regression for continuous variables and logistic regression for categorical variables, adjusting for sex, age, and country as well as baseline accelerometer wear time and season for accelerometer variables. When examining differences in BMI between dropouts and completers, screening data were used rather than baseline data because 38% of dropouts had no baseline data. R software version 3.1.2 [23] was used to perform all analyses and the significance level was set at *P*<.05.

## Results

###  Study Participants

**Figure 1:**
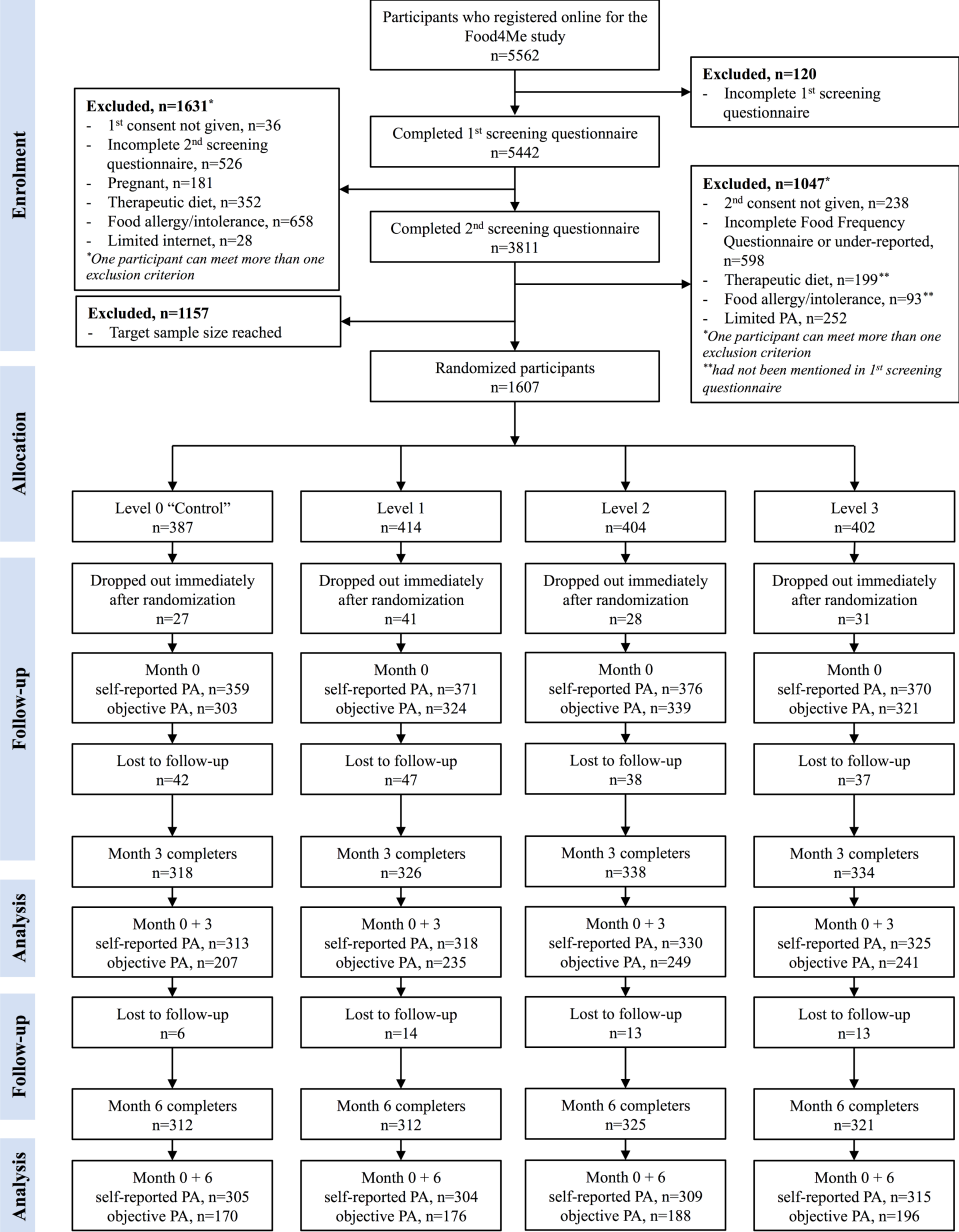
Flow of participants through the study. PA: physical activity.

Of the 5562 individuals who expressed an interest in the Food4Me Study between August 2012 and August 2013, 4044 (72.71%) completed the whole screening process (Figure 1). Of those, 2764 (68.35%) were eligible to take part in the intervention study. The first 1607 of the 2764 (58.14%) participants were randomized to one of the four intervention arms and 127 (7.90%) dropped out immediately after randomization (Figure 1).

The characteristics of the 1480 participants who started the trial and completed baseline measurements are given by intervention arm in Table 1 and Multimedia Appendix 3, and are described elsewhere [18]. Overall, 58.45% (865/1480) were women, the mean age was 39.9 (SD 13.0) years, and 46.15% (683/1480) of participants were overweight or obese. Mean PAL was 1.73 (SD 0.18), and participants spent on average 12.4 (SD 1.3) h/d in sedentary behaviors and 57 (SD 45) min/d in moderate-equivalent PA (29 [SD 32] min/d in modified 10-minute bouts). Mean self-reported total activity index was 7.80 (SD 1.48) (Table 1). Of the 371 participants in L3, 257 (69.3%) and 113 (30.5%) were carriers of the risk (AA or AT) and nonrisk (TT) genotypes for *FTO*, respectively (Table 1), and were therefore informed that they had or did not have the risk variant. A total of 807 of 1120 (72.05%) individuals randomized to the personalized groups (L1 to L3) were not sufficiently active based on baseline measurements and therefore received feedback that they should increase their level of PA (data not shown).

**Table 1:** Baseline characteristics^a^ of the Food4Me participants.

Variables		Control	Personalized advice		
		Level 0 (L0) (n=360)	Level 1 (L1) (n=373)	Level 2 (L2) (n=376)	Level 3 (L3) (n=371)
Ethnicity (white), n (%)		344 (95.6)	363 (97.3)	368 (97.9)	357 (96.2)
Sex (women), n (%)		213 (59.2)	212 (56.8)	220 (58.5)	220 (59.3)
Age (years), mean (SD)		39.5 (13.3)	39.7 (12.9)	40.2 (12.8)	40.2 (13.1)
**Anthropometrics**					
	Height (cm), mean (SD)	171.3 (9.4)	171.3 (9.5)	170.7 (9.4)	171.2 (9.5)
	Weight (kg), mean (SD)	74.6 (15.5)	74.1 (16.6)	74.9 (15.9)	75.5 (15.5)
	Body mass index (kg/m^2^), mean (SD)	25.4 (4.7)	25.2 (5.0)	25.6 (4.9)	25.7 (4.8)
	Overweight, n (%)	119 (33.1)	96 (25.7)	103 (27.4)	131 (35.3)
	Obese, n (%)	52 (14.4)	57 (15.3)	70 (18.6)	55 (14.8)
	Current smokers, n (%)	49 (13.6)	44 (11.8)	34 (9.0)	47 (12.7)
	Ex-smokers, n (%)	88 (24.4)	98 (26.3)	101 (26.9)	91 (24.5)
	Nonsmokers, n (%)	223 (61.9)	231 (61.9)	241 (64.1)	233 (62.8)
***FTO* ^b^ genotype, n (%)**					
	AA	60 (16.7)	69 (18.5)	66 (17.6)	69 (18.6)
	AT	187 (51.9)	175 (46.9)	189 (50.3)	188 (50.7)
	TT	112 (31.1)	127 (34.0)	117 (31.1)	113 (30.5)
**Objective physical activity (PA)** **(n_L0_=303, n_L1_=324, n_L2_=339, n_L3_=321), mean (SD)**					
	Physical activity level	1.71 (0.18)	1.75 (0.21)	1.73 (0.16)	1.74 (0.17)
	Activity energy expenditure (kcal/d)	832 (269)	896 (312)	869 (274)	874 (283)
	Sedentary time (min/d)	746 (76)	738 (75)	747 (78)	749 (77)
	Light-intensity PA (min/d)	70 (27)	76 (33)	74 (31)	76 (30)
	Moderate-intensity PA (min/d)	30 (19)	35 (20)	33 (21)	34 (22)
	Vigorous-intensity PA (min/d)	11 (16)	14 (20)	11 (14)	10 (14)
	Moderate-equivalent PA^c^ (min/d)	53 (43)	63 (50)	56 (42)	55 (45)
	Moderate-equivalent PA in bouts (min/d)	27 (32)	34 (38)	28 (28)	28 (30)
**Self-reported PA** **(n_L0_=359, n_L1_=371, n_L2_=376, n_L3_=370), mean (SD)**					
	Total activity index	7.71 (1.47)	7.94 (1.48)	7.78 (1.43)	7.80 (1.54)
	Work index	2.26 (0.59)	2.30 (0.61)	2.28 (0.62)	2.29 (0.62)
	Sport index	2.70 (0.87)	2.85 (0.89)	2.73 (0.85)	2.74 (0.88)
	Leisure time (nonsport) index	2.75 (0.69)	2.81 (0.70)	2.78 (0.69)	2.78 (0.67)

^a^Data are presented as unadjusted means (SD) for continuous variables and absolute numbers (%) for categorical variables. Levels 1 to 3 received personalized advice; only participants in Level 3 were informed whether they carried or did not carry the risk allele for *FTO* (A).

^b^Fat mass and obesity associated (*FTO*).

^c^Moderate-equivalent PA is (MPA + 2 × VPA).

Of the 1607 randomized participants, 1270 (79.03%) completed the 6-month intervention and 1233 (76.73%) had self-reported data on PA for both baseline and month 6, whereas only 730 (45.43%) had sufficient valid accelerometer data for both time points (Figure 1). Dropouts were more likely to be women (odds ratio [OR] 1.34, 95% CI 1.05-1.75, *P*=.03), were younger than completers (*P*<.001), and had a higher BMI at screening than completers (*P*=.02). Smoking habits did not differ significantly between dropouts and completers, and the dropout rate was similar in all four groups, L0 to L3 (data not shown). Among completers, those who were not compliant with objective PA measurement at month 6 were younger (*P*<.001), had a higher baseline BMI (*P*=.04), and a lower baseline PAL (*P*=.03). There were no significant differences in smoking habits between those who were compliant and those who were not, and compliance was similar for men and women and in all four groups, L0 to L3 (data not shown).

### Effect of Different Levels of Personalized Advice on Objective Physical Activity

#### Total Cohort: Generic Approach

At month 3 (see Multimedia Appendix 4), participants increased their PAL (L0: +0.02, *P*=.008 and L1 to L3: +0.02, *P*<.001) and AEE (L0: +24.2 kcal/d, *P*=.03 and L1 to L3: +19.5 kcal/d, *P*=.001), and spent significantly more time in MPA (L0: +18 min/wk, *P*=.01 and L1 to L3: +17 min/wk, *P*<.001) and less time in sedentary behavior (L0: -148 min/wk, *P*<.001 and L1 to L3: -133 min/wk, *P*<.001). No significant change in PA was observed at month 6 (see Multimedia Appendix 5), except for a significant decrease in sedentary time (L0: -190 min/wk, *P*<.001 and L1 to L3: -155 min/wk, *P*<.001). Furthermore, we found no significant differences in objectively measured PA between control and personalized groups, or between personalized groups, at month 3 or 6 (Multimedia Appendices 4 and 5).

#### Participants Who Received Advice to Increase Physical Activity and Matched Controls: Targeted Approach

**Figure 2:**
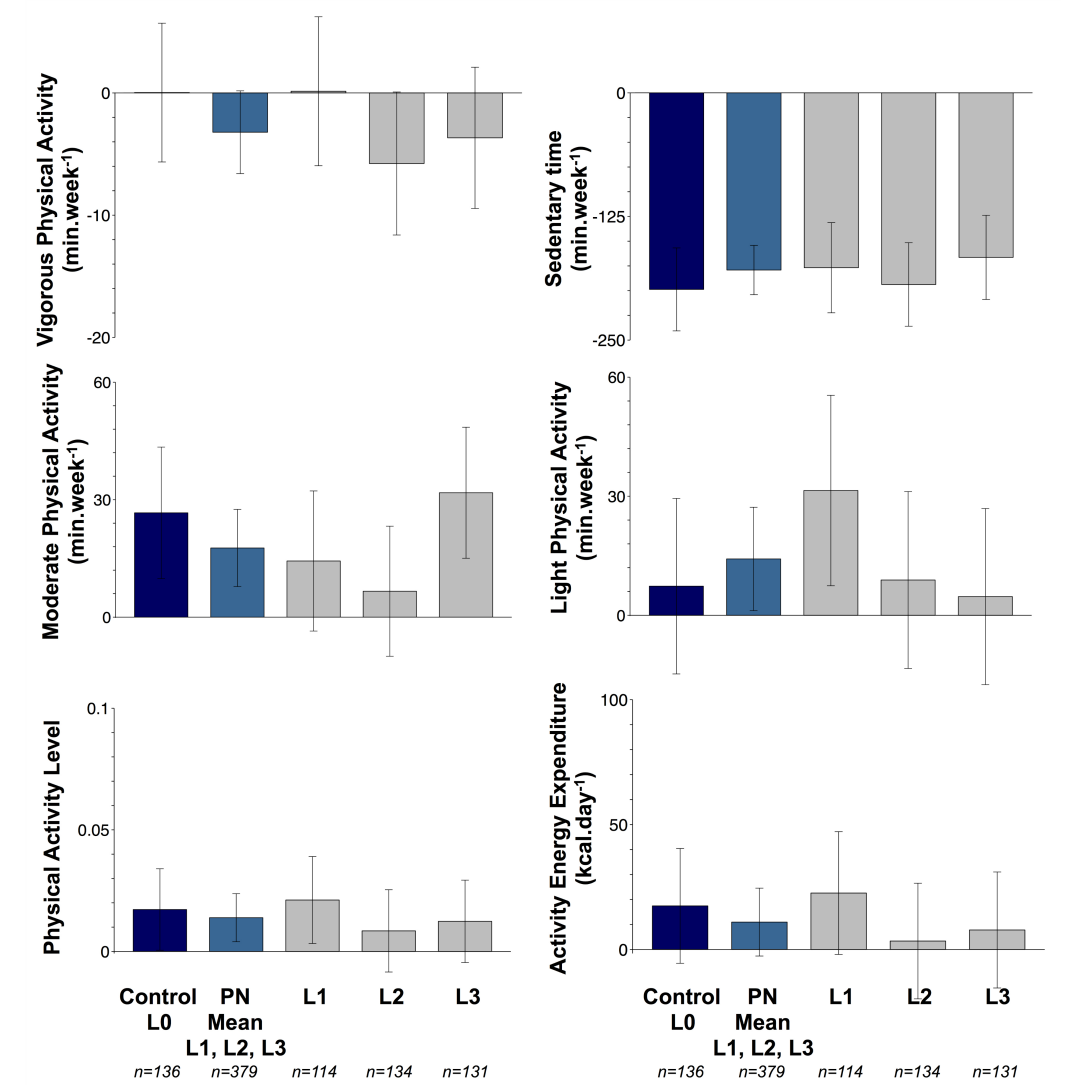
Changes from baseline to month 6 in physical activity measured objectively, for participants who received advice to increase physical activity (personalized groups Levels 1, 2, and 3) and matched controls (Level 0)—targeted approach. Data are presented as adjusted changes from baseline. Error bars represent 95% confidence intervals. Models were adjusted for baseline values, sex, age, country, smoking, baseline BMI, baseline season, change in body weight, and change in accelerometer wear time. Individuals in Levels 1 (L1), 2 (L2), and 3 (L3) received personalized physical activity feedback based on current physical activity level (L1 to L3), phenotypic information (L2 and L3), and genotypic information (L3), whereas controls (L0) received nonpersonalized guidelines on physical activity.

At month 3, we observed significant improvements in all components of PA for participants in personalized groups as well as for matched controls (see Multimedia Appendix 6). Although changes were attenuated at month 6 (Figure 2) these remained significant for PAL (L0: +0.02, *P*=.04 and L1 to L3: +0.01, *P*=.006), sedentary time (L0: -199 min/wk, *P*<.001 and L1 to L3: -179 min/wk, *P*<.001), and MPA (L0: +27 min/wk, *P*=.002 and L1 to L3: +18 min/wk, *P*<.001).

However, there were no significant differences in objectively measured PA between individuals in personalized groups and matched controls (Table 2 and Figure 2).

At month 6, the change from baseline in MPA was significantly larger for L3 compared with L2 (L3: +32 min/wk vs L2: +7 min/wk, *P*=.04), but there were no other significant differences in PA between personalized groups (see Figure 2 and Multimedia Appendix 7). Results were unchanged when analyzing men and women separately (data not shown).

### Effect of Different Levels of Personalized Advice on Self-Reported Physical Activity

#### Total Cohort: Generic Approach

Participants reported significant improvements in PA after the 6-month intervention (see Multimedia Appendix 8). Compared with the control group, individuals in personalized groups had significantly higher leisure time (nonsport) index (2.4%, *P*=.02) and total activity index scores (1.6%, *P*=.03) (see Multimedia Appendix 8). However, no significant differences were found between personalized groups (see Multimedia Appendix 8). Similar results were found at month 3 (see Multimedia Appendix 9).

#### Participants Who Received Advice to Increase Physical Activity and Matched Controls: Targeted Approach

**Figure 3:**
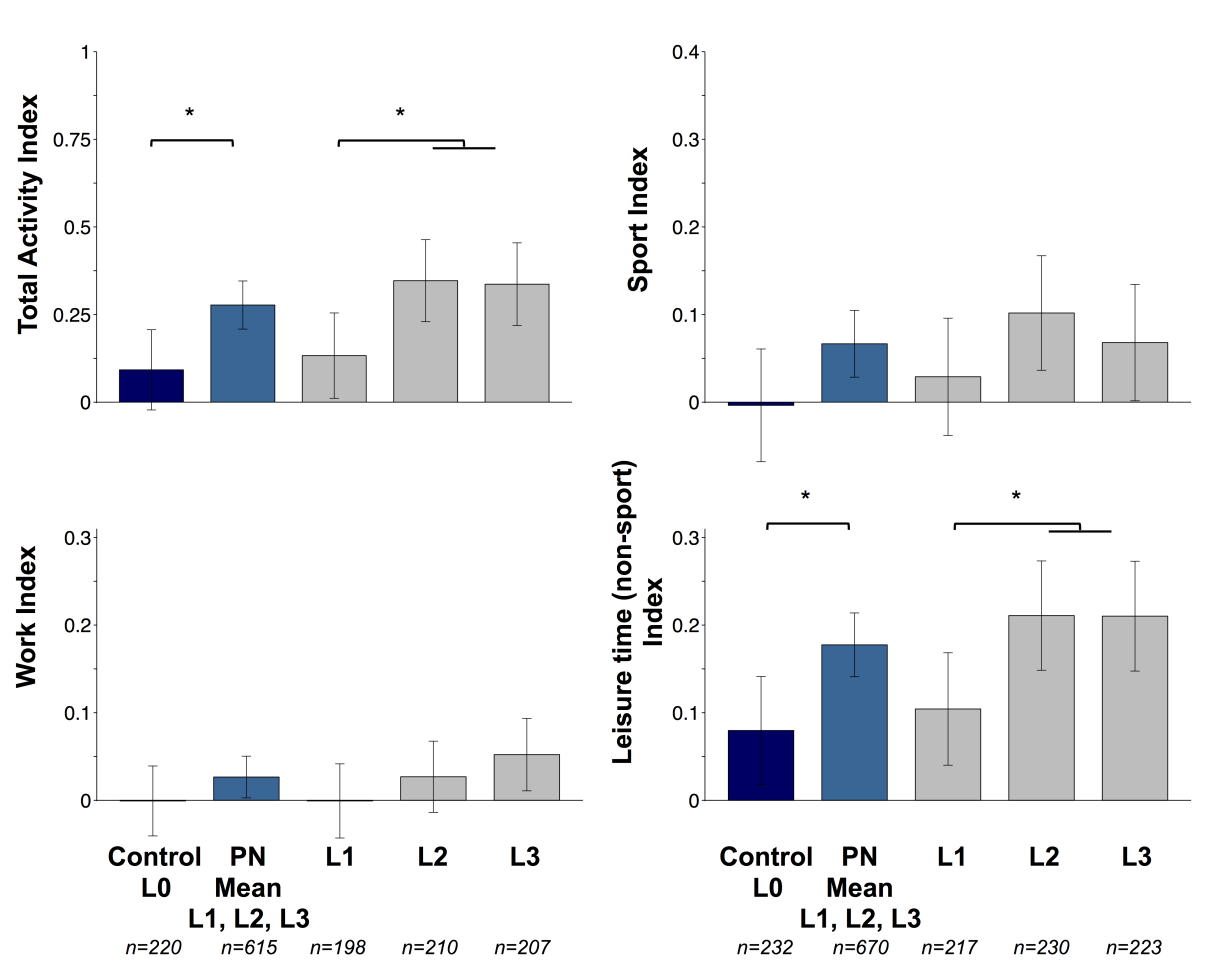
Changes from baseline to month 6 in self-reported physical activity (Baecke questionnaire) for participants who received advice to increase physical activity (personalized groups Levels 1, 2, and 3) and matched controls (Level 0)—targeted approach. Data are presented as adjusted changes from baseline. Error bars represent 95% confidence intervals. Models were adjusted for baseline values, sex, age, country, smoking, baseline BMI, baseline season, and change in body weight. *Significant differences at P<.01. Individuals in Levels 1 (L1), 2 (L2), and 3 (L3) received personalized physical activity feedback based on current physical activity level (L1 to L3), phenotypic information (L2 and L3), and genotypic information (L3), whereas controls (L0) received nonpersonalized guidelines on physical activity.

After 6 months, there were significant improvements in self-reported PA during sport, leisure time (nonsport), and total PA among participants who received tailored advice (Figure 3). Compared with the control group, scores reported in personalized groups were significantly higher for leisure time (nonsport) index (3.6%, *P*=.009) and total activity index (2.5%, *P*=.009) (see Table 2 and Figure 3). Similar results were found at month 3 (see Multimedia Appendix 10). Finally, we also observed significant differences between personalized groups at month 6, scores for both indices being higher (3.9%, *P*=.006 and 2.9%, *P*=.008, respectively) for participants in L2 and L3 compared with L1 (see Figure 3 and Multimedia Appendix 7). Results were unchanged when analyzing men and women separately.

Importantly, results were also similar when including only individuals with both objective and self-reported PA (ie, completers, compliant with wearing the accelerometer and who have self-reported data) in the analysis (data not shown).

**Table 2:** Effect of targeted intervention on physical activity at month 6^a^.

PA^b^ components		Matched control, L0^c^ (n=136), mean (SD)	Personalized advice, L1 to L3^d^ (n=379), mean (SD)	Intervention effects, (L1 to L3) - L0 (95% CI)	*P,* L0 vs (L1 to L3)
**Objective PA**					
	PAL^e^	1.68 (0.10)	1.68 (0.10)	-0.003 (-0.020 to 0.020)	.73
	AEE^f^ (kcal/d)	785 (137)	778 (135)	-6 (-34 to 21)	.64
	Sedentary time (min/wk)	5182 (250)	5202 (247)	20 (-30 to 69)	.44
	LPA^g^ (min/wk)	479 (132)	486 (129)	7 (-19 to 33)	.59
	MPA^h^ (min/wk)	216 (100)	207 (98)	-9 (-29 to 11)	.36
	VPA^i^ (min/wk)	48 (34)	45 (34)	-3 (-10 to 3)	.33
	Moderate-equivalent PA^j^ (min/wk)	323 (154)	310 (154)	-14 (-45 to 17)	.35
	Moderate-equivalent PA in bouts (min/wk)	140 (103)	131 (102)	-10 (-30 to 11)	.35
**Self-reported PA**					
	Total activity index^k^	7.58 (0.87)	7.77 (0.87)	0.18 (0.05 to 0.32)	*.009* ^l^
	Work index^k^	2.24 (0.30)	2.27 (0.30)	0.03 (-0.02 to 0.07)	.26
	Sport index^m^	2.58 (0.50)	2.65 (0.50)	0.070 (-0.005 to 0.150)	.07
	Leisure time (nonsport) index^m^	2.77 (0.48)	2.87 (0.48)	0.10 (0.03 to 0.17)	*.009*

^a^Analysis is restricted to participants randomized to Levels 1 to 3 (L1, L2, and L3) who received personalized advice to increase PA, and to matched control group (L0) participants who would have received personalized advice to increase PA if they had been in a personalized group and not in L0. Data are presented as adjusted means and as the difference between the personalized groups (mean L1, L2, L3) and control with the corresponding 95% confidence interval. Differences between levels of personalized advice are presented in Multimedia Appendix 7. All analyses were adjusted for baseline values, sex, age, country, smoking, baseline BMI, baseline season, and change in body weight. In addition, for objective PA variables analyses were adjusted for change in accelerometer wear time.

^b^Physical activity (PA).

^c^Level 0 (L0).

^d^Level 1 to Level 3 (L1 to L3).

^e^Physical activity level (PAL).

^f^Activity energy expenditure (AEE).

^g^Light-intensity PA (LPA).

^h^Moderate-intensity PA (MPA).

^i^Vigorous-intensity PA (VPA).

^j^Moderate-equivalent PA is (MPA + 2 × VPA).

^k^Participant numbers within each group are as follows: n=220 (L0), 198 (L1), 210 (L2), 207 (L3), and 615 (pooled L1, L2, and L3). For retired or unemployed individuals, work index, and therefore total index, cannot be calculated.

^l^Values in italics represent significant results.

^m^Participant numbers within each group are as follows: n=232 (L0), 217 (L1), 230 (L2), 223 (L3), and 670 (pooled L1, L2, and L3).

## Discussion

### Principal Findings

For individuals who were not sufficiently active at baseline, as well as for the total cohort, personalized PA advice delivered via the Internet was more effective in improving self-reported PA compared with conventional “one size fits all” population-based advice. In addition, after 6 months of intervention, including phenotypic and/or genotypic information in the derivation of personalized PA advice led to bigger changes in self-reported PA than personalization based on diet and PA alone. However, these findings were not confirmed when PA was assessed objectively using accelerometers. Although we found some small significant improvements in objectively measured PA over the 6-month intervention, these changes were similar in all interventions groups.

### Comparison With Previous Work

Most studies that have investigated the effectiveness of eHealth computer-tailored PA interventions have relied on self-reports [8], and their findings should be interpreted with caution. Few studies were identified that used objective PA outcome measures based on accelerometry [14-17,32]. Godino et al [15] noted that personalized PA feedback increased awareness of PA but did not promote change in PA after 2 months of follow-up. However, Hurling et al [32] found that participants who had access to a fully automated Internet, email, and mobile phone behavior change system—which included feedback on activity level and modules designed to help participants identify their perceived barriers and offered tailored solutions—had significantly higher objectively measured PA during the 9-week intervention compared with controls, who received verbal recommendations on PA but had no access to the behavior change system and received no feedback. Self-reported leisure PA was also significantly higher and time spent sitting significantly lower in the intervention group compared with the controls, but overall self-reported PA was similar in both groups [32]. In a study of older adults, Wijsman et al [17] observed that participants in an Internet program aiming to increase PA by monitoring and feedback by accelerometer and digital coaching had a significant increase of 11 minutes per day in moderate and vigorous PA after 3 months, whereas the wait-listed controls showed no change in PA. Ashe et al [14] found significant increases in PA, as well as group differences at month 6, but their sample was small—13 participants in the intervention group and 12 in the control group—and group-based education and social support was included in addition to their Web-based intervention. Finally, Wanner et al [16] reported some improvements in self-reported PA after 6 weeks and 13 months of follow-up, but no differences between individuals in tailored and control groups, and no improvement in objectively measured PA for any group. These discrepancies between self-reported and objectively measured PA results are in line with our study. However, we found greater improvements in self-reported PA in tailored groups as compared with the controls. It could be that participants desired to comply with recommendations and that receiving more personalized feedback (Levels 2 and 3) increased this desire further in our study. It could also be that participants truly believed that they became more active when they actually did not. Contrary to Wanner et al, objective PA also improved, slightly but significantly, after 6 months of intervention, especially in participants who were inactive at baseline, in line with the results of Wijsman et al and Ashe et al. Yet in our study, those changes in objective PA were similar across all groups.

The number of studies testing the effectiveness of personalized feedback versus conventional population-based guidelines is limited. However, most authors stress the need for new ways to increase compliance and engagement of participants to ultimately successfully improve PA. Of those who started the Food4Me Study, 85.81% (1270/1480) completed the trial, indicating that our Web-based intervention was effective in retaining participants. However, only 49.32% (730/1480) of the starters had sufficient valid accelerometer data after 6 months. Thus, compliance with the study protocol in wearing the accelerometer remains a major issue, especially because those who were less compliant in our study had significantly lower baseline PAL.

### Strengths and Limitations

The Food4Me Study is the largest Internet-based RCT to date to test the effects of personalized feedback on PA, using objectively measured PA with accelerometers. To our knowledge, it is also the first study to assess the effects of different levels of personalization including tailored phenotypic and genotypic information. Another strength is the inclusion of seven European centers that delivered the intervention with the same standardized protocol [18,33].

An important limitation is the relatively low compliance after 6 months with respect to accelerometer wear, which is lower than in other studies [16]. In our study, participants were asked to wear their accelerometer every day for the entire duration of the study, which may have been too demanding. Most participants did wear the accelerometer but not enough (ie, fewer than 3 valid weekdays and 2 valid weekend days). In other studies, participants received a PA monitor, were asked to wear it for the measurement period only (typically, 7 days), and to return the device immediately after to the researchers [15-17]. Better compliance in wearing the monitor may be obtained by having coaches motivating participants regularly [17], but this is not always feasible in a large-scale study. Advertisement for the study was primarily focused on personalized nutrition (ie, improving nutritional intakes) and not on PA. Moreover, the Food4Me Study was a multiple-behavior intervention including a large amount of information with extensive feedback, and many individuals may have felt they did not have time to try to make changes in both PA and diet concurrently [34].

A potential explanation as to why participants in personalized groups did not do better than controls could be that the TracmorD PA monitor used in the study constituted a basic, yet personalized, feedback by itself. That is, when the monitor is set on a flat surface at any time (eg, a table), lights on the monitor turn green depending on how much activity has been registered during the day. The more activity, the more lights turn green, for all participants including controls. Ideally, there should be no feedback on PA at all in the control group. This may explain the small but significant improvement in the control group. One could argue that providing feedback every 3 months might not be sufficiently frequent. In our study, half of the participants in the personalized groups received additional feedback based on measurements after 1 and 2 months (ie, high-intensity feedback: four feedback reports at months 0, 1, 2, and 3) but changes in objectively and self-reported PA at 3- and 6-month follow-up were similar to the other half of participants (ie, low-intensity feedback: feedback reports at months 0 and 3 only). Compliance in wearing the monitor did not differ between high- and low-intensity participants (data not shown). Furthermore, we cannot exclude the fact that phenotypic and genotypic characterization, and therefore feedback to participants in Levels 2 and 3, may not have been optimal for PA-related outcomes. For example, our only PA-related genotypic variant was in the *FTO* gene and perhaps just one gene variant would be insufficient to motivate participants in Level 3 to increase PA beyond those in Level 2.

Finally, although accelerometry is an objective measure of PA, it can underestimate certain activities, such as carrying heavy loads and when the torso remains relatively static (eg, during cycling). Accelerometry cannot (easily) distinguish PA when ascending (eg, walking uphill) from movement on the flat yet there could be large differences in energy expenditure between the two types of movement. Our monitor was waterproof and could be worn during swimming, but underestimation of activity intensity is common. Nevertheless, the TracmorD has been validated against the doubly labeled water method and several publications show that it is a reliable and accurate monitor [12,13,20,35]. Although devices may not capture all types of PA, questionnaires have been shown repeatedly to be inaccurate, often overestimating PA [11,36]. The Baecke questionnaire, although extensively validated [28,29], is no exception [37]. Our results support the position adopted by others that self-reported measures of PA should be interpreted with caution and preferably not be used to draw conclusions on the effectiveness of PA interventions [8,38]. Thus, it is better for personalized feedback to have objective measures of PA such as accelerometry. Such technologies are becoming very relevant tools for both surveys and interventions to promote public health. They are developing rapidly and are commonly available for download to mobile phones and watches, allowing greater accessibility for the general population, which may help with noncompliance. However, these new apps will need to be rigorously tested.

### Conclusions

We observed small but significant improvements in objectively measured PA after 3 and 6 months of intervention, although changes were similar across all groups. Personalized advice on PA did not promote larger and more sustained improvements in objective PA as compared with a conventional “one size fits all” approach delivered via the Internet. Furthermore, increasing the degree of personalization using phenotypic or genotypic information had no effect on changes in objectively measured PA compared with personalized feedback based on diet and PA alone. Based on self-reports, however, PA improved significantly more with higher degrees of personalized advice.

## Appendix

Multimedia Appendix 1 CONSORT-EHEALTH checklist V1.6.1 [39].

Multimedia Appendix 2 Physical activity in feedback reports and on the Food4Me website.

Multimedia Appendix 3 Baseline characteristics of the Food4Me participants who received personalized advice to increase physical activity and matched controls—targeted approach.

Multimedia Appendix 4 Changes from baseline to month 3 in objectively measured physical activity for the total cohort—generic approach. Data are presented as adjusted changes from baseline. Error bars represent 95% confidence intervals. Models were adjusted for baseline values, sex, age, country, smoking, baseline BMI, baseline season, change in body weight, and change in accelerometer wear time.

Multimedia Appendix 5 Changes from baseline to month 6 in objectively measured physical activity for the total cohort—generic approach. Data are presented as adjusted changes from baseline. Error bars represent 95% confidence intervals. Models were adjusted for baseline values, sex, age, country, smoking, baseline BMI, baseline season, change in body weight, and change in accelerometer wear time.

Multimedia Appendix 6 Changes from baseline to month 3 in objectively measured physical activity for participants who received advice to increase PA (L1 to L3) and matched controls (L0)—targeted approach. Data are presented as adjusted changes from baseline. Error bars represent 95% confidence intervals. Models were adjusted for baseline values, sex, age, country, smoking, baseline BMI, baseline season, change in body weight, and change in accelerometer wear time.

Multimedia Appendix 7 Effect of targeted intervention on physical activity for personalized groups at month 6.

Multimedia Appendix 8 Changes from baseline to month 6 in self-reported physical activity (Baecke questionnaire) for the total cohort—generic approach. Data are presented as adjusted changes from baseline. Error bars represent 95% confidence intervals. Models were adjusted for baseline values, sex, age, country, smoking, baseline BMI, baseline season, and change in body weight.

Multimedia Appendix 9 Changes from baseline to month 3 in self-reported physical activity (Baecke questionnaire) for the total cohort—generic approach. Data are presented as adjusted changes from baseline. Error bars represent 95% confidence intervals. Models were adjusted for baseline values, sex, age, country, smoking, baseline BMI, baseline season, and change in body weight.

Multimedia Appendix 10 Changes from baseline to month 3 in self-reported physical activity (Baecke questionnaire) for participants who received advice to increase physical activity (L1 to L3) and matched controls (L0)—targeted approach. Data are presented as adjusted changes from baseline. Error bars represent 95% confidence intervals. Models were adjusted for baseline values, sex, age, country, smoking, baseline BMI, baseline season, and change in body weight.

## Abbreviations

AEE: activity energy expenditure
BMI: body mass index
BMR: basal metabolic rate
CIBERobn: Centro de Investigación Biomédica en Red-Fisiopatología de la Obesidad y Nutrición
FTO: fat mass and obesity associated
GP: general practitioner
IPAQ: International Physical Activity Questionnaire
IZZ: National Food & Nutrition Institute
KASP: competitive allele-specific polymerase chain reaction
L0: Level 0
L1: Level 1
L2: Level 2
L3: Level 3
LPA: light-intensity physical activity
MET: metabolic equivalent
MPA: moderate-intensity physical activity
MUMC+: Maastricht University Medical Centre +
NUTRIM: (School of) Nutrition and Translational Research in Metabolism
OR: odds ratio
PA: physical activity
PAL: physical activity level
RCT: randomized controlled trial
SNP: single nucleotide polymorphism
UCD: University College Dublin
VPA: vigorous-intensity physical activity
WHO: World Health Organization
ZIEL: Zentralinstitut für Ernährungs- und Lebensmittelforschung
