# Supplementary material for: Effects of a Web-Based Personalized Intervention on Physical Activity in European Adults: A Randomized Controlled Trial
Source: J Med Internet Res. 2015 Oct 14;17(10):e231. doi: 10.2196/jmir.4660 (PMC4642412; doi:10.2196/jmir.4660)
Supplement: Multimedia Appendix 2 [file jmir_v17i10e231_app2.pdf]

Feedback on physical activity in personalized reports

Based on the body measurements and physical activity that you recorded, your physical characteristics have been rated below:

Your score

Your height: 1.72 m

Your weight: 89 kg

Your BMI: 30.1 kg/m^2

Underweight	Healthy	Overweight	Obese
<18.5	18.5-24.9	25-29.9	>30

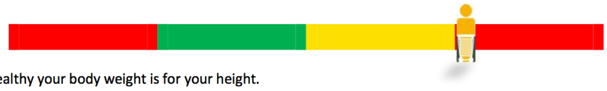

BMI = Body Mass Index. This is an indicator of how healthy your body weight is for your height.

Your waist circumference: 92 cm

Healthy	Above recommended
< 88 cm	> 88 cm

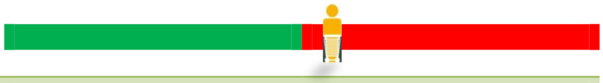

Your Physical Activity level:

Improvement strongly recommended	Improvement recommended	Good, keep up the good work

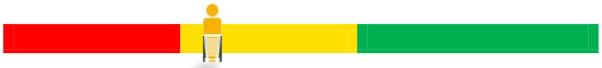

Section 4: Your Personalised Nutrition Advice

Your Weight and Physical Activity Recommendations

Your BMI is greater than the recommended healthy range, indicating that you are very overweight for your height. Your waist circumference is also higher than recommended. Carrying too much weight around your middle increases your risk of certain diseases including heart disease and cancer. We recommend reducing your body weight and waist circumference to a healthy normal range because you have a genetic variation that can benefit by reducing these two obesity markers. We strongly recommend that you try to reduce your weight; a weight loss of 0.5-1.0kg (1-2lbs) a week is a realistic goal. Although your physical activity level is good, increasing it will help you to reduce your weight. Your fasting cholesterol level was also slightly above the recommended levels.

The following list contains suggestions to help you to lose weight:

•Increase your physical activity; to maintain weight loss, 60-90 minutes of moderately intense aerobic activities, such as brisk walking, swimming or cycling, on most days of the week, is recommended. This also helps to lower cholesterol levels.

•Reduce your portion sizes

•Eat regularly and avoid skipping meals

•Avoid snacking on foods high in sugar and fat - swap these for healthier alternatives, such as fruit

•Choose low-fat options

Information on physical activity and tips on the Food4Me webpage for participants in personalized nutrition (PN) groups

Extranet | Food4Me

dopis_hance@yahoo.co.uk

Welcome back Hana

Edit Details | Log Out

Home Instructions Info Physical Activity FAQ About

Physical Activity: Crucial For A Healthy Lifestyle

The importance of physical activity for health

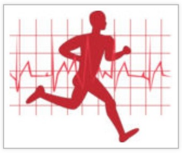

Physical activity is an essential element within a healthy lifestyle pattern. If you combine healthy, varied and balanced dietary habits with the regular practice of moderate exercise, you should be able to prevent most of cardiovascular diseases. In addition, you will manage to keep a healthy and adequate body weight.

We can divide physical activity into three categories:

- Stretching: Exercises focused on giving flexibility to muscles and joints.
- Aerobic: Exercise focused on increasing cardiovascular endurance
- Anaerobic: Exercise focused on increasing short-term muscle strength.

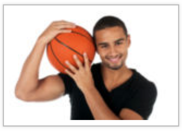

Aerobic exercise is the type of exercise with which our health will improve, and it can be graded depending on your age and physical conditions. Examples of this type of exercise are fast walking, soft running or jogging, tennis practice, swimming, etc.

Research has focused on how exercise can improve your health, and to which extent physical activity is important to help to prevent chronic diseases. The regular practice of aerobic exercise of moderate intensity has been proven to help maintain body weight, by reducing fat mass and increasing muscle strength, to build and maintain bone density, strengthen the immune system, and reduce surgical risks. It also helps to promote a physiological and psychological well-being. Aerobic exercise has also been shown to help prevent or treat serious and life-threatening chronic conditions such as high blood pressure, heart disease, Type 2 diabetes, insomnia, and depression.

According to the World Health Organization, lack of physical activity contributes to approximately 17% of heart disease and diabetes, 12% of falls in the elderly, and 10% of breast and colon cancer.

How physically active should you be?

You need at least:

2 hours and 30 minutes (150 minutes) of **moderate-intensity aerobic activity** (ie. brisk walking) every week

\+

Muscle-strengthening activities on 2 or more days a week that work all major muscle groups (legs, hips, back, abdomen, chest, shoulders and arms)

OR

1 hour and 15 minutes (75 minutes) of **vigorous-intensity aerobic activity** (ie. jogging/running) every week

\+

Muscle-strengthening activities on 2 or more days a week that work all major muscle groups (legs, hips, back, abdomen, chest, shoulders and arms)

OR

An equivalent mix of **moderate and vigorous-intensity aerobic activity**

\+

Muscle-strengthening activities on 2 or more days a week that work all major muscle groups (legs, hips, back, abdomen, chest, shoulders and arms)

Tips for improving your physical activity level

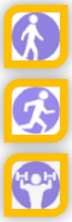

10 minutes at a time is fine!
We know 150 minutes each week sounds like a lot of time, but you don't have to do it all at once. Not only is it best to spread your activity out during the week, but you can break it up into smaller chunks of time during the day. As long as you're doing your activity at a moderate or vigorous effort for at least 10 minutes at a time.

Give it a try!
Try going for a 10 minute brisk walk, 3 times a day, 5 times a week. This will give you a total of 150 minutes of moderate intensity physical activity.

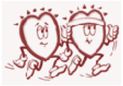

Remember that being active...

- Reduces risk of obesity, cardiovascular diseases, diabetes
- Helps to maintain a healthy weight
- Helps to maintain the ability to perform everyday tasks with ease
- Improves self-esteem
- Reduces symptoms of depression and anxiety

Moderate intensity aerobic activity

means you're working hard enough to raise your heart rate and start to sweat. One way to tell is that you'll be able to talk, but not sing your favourite song. Here are some examples of activities which require a moderate effort:

- Walking fast
- Doing water aerobics
- Riding a bike on level ground or with few hills
- Playing doubles tennis
- Pushing a lawnmower

Vigorous intensity aerobic activity

means that you're breathing hard and fast, and your heart rate has gone up quite a bit. If you're active at this level, you won't be able to say more than a few words without pausing to catch your breath. Here are some examples of activities which require a vigorous effort:

- Jogging or running
- Swimming laps
- Riding a bike fast or on hills
- Playing singles tennis
- Playing basketball
