# Supplementary material for: Effects of a Web-Based Personalized Intervention on Physical Activity in European Adults: A Randomized Controlled Trial
Source: J Med Internet Res. 2015 Oct 14;17(10):e231. doi: 10.2196/jmir.4660 (PMC4642412; doi:10.2196/jmir.4660)
Supplement: Multimedia Appendix 3 [file jmir_v17i10e231_app3.pdf]

**Variables**	**Matched Control**	**Personalized advice**		
	Level 0	Level 1	Level 2	Level 3
	n=275	n=268	n=274	n=265
Ethnicity - White (%)	94.9	97	98.2	96.6
Sex - Women (%)	62.2	56	60.6	63.4
Age (years)	40.2 (13.2)	39.4 (13.1)	40.8 (12.9)	40.1 (13.2)
**Anthropometrics**				
Height (cm)	170.9 (9.4)	171.9 (9.6)	170.4 (9.3)	170.5 (9.2)
Weight (kg)	74.8 (16)	75.4 (17.6)	75.4 (16)	76.1 (16.3)
BMI (kg.m^{-2})	25.6 (5)	25.5 (5.4)	25.9 (5.1)	26.1 (5.1)
Overweight (%)	32.4	26.5	27.4	37.7
Obese (%)	16.4	17.5	21.2	17.7
Current smokers (%)	14.9	13.4	9.5	14.7
Ex-smokers (%)	22.9	26.1	24.8	23
Non-smokers (%)	62.2	60.4	65.7	62.3
**Objective PA**				
	n=237	n=233	n=246	n=230
PAL	1.64 (0.09)	1.66 (0.11)	1.66 (0.11)	1.66 (0.1)
AEE (kcal.d^{-1})	735 (178)	782 (215)	769 (191)	759 (182)
Sedentary time (min.d^{-1})	757 (73)	747 (78)	757 (73)	763 (73)
LPA (min.d^{-1})	65 (24)	68 (28)	66 (24)	66 (24)
MPA (min.d^{-1})	26 (16)	30 (17)	27 (16)	27 (17)
VPA (min.d^{-1})	6 (9)	8 (10)	7 (10)	6 (8)
Moderate-equivalent PA (min.d^{-1})	38 (28)	46 (32)	42 (29)	39 (27)
Moderate-equivalent PA in bouts (min.d^{-1})	16 (19)	20 (21)	19 (18)	18 (19)
**Self-reported PA**				
	n=275	n=268	n=274	n=264
Total activity index	7.32 (1.25)	7.64 (1.36)	7.49 (1.3)	7.35 (1.4)
Work index	2.21 (0.56)	2.3 (0.62)	2.23 (0.58)	2.22 (0.59)
Sport index	2.49 (0.75)	2.63 (0.79)	2.59 (0.8)	2.53 (0.8)
Leisure time (non-sport) index	2.63 (0.65)	2.73 (0.65)	2.67 (0.64)	2.64 (0.64)

Data are presented as unadjusted means (SD) for continuous variables and percentages for categorical variables. Levels 1 – 3 received personalized advice. Matched control group (Level 0) participants are controls who would have received personalized advice to increase PA if they had been in a PN group and not L0. PA, physical activity; PAL, physical activity level; AEE, activity energy expenditure; LPA, light PA; MPA, moderate PA; VPA, vigorous PA; Moderate-equivalent PA is (MPA + 2 × VPA).
