# Supplementary figures and images for: Effects of a Web-Based Personalized Intervention on Physical Activity in European Adults: A Randomized Controlled Trial
Source: J Med Internet Res. 2015 Oct 14;17(10):e231. doi: 10.2196/jmir.4660 (PMC4642412; doi:10.2196/jmir.4660)

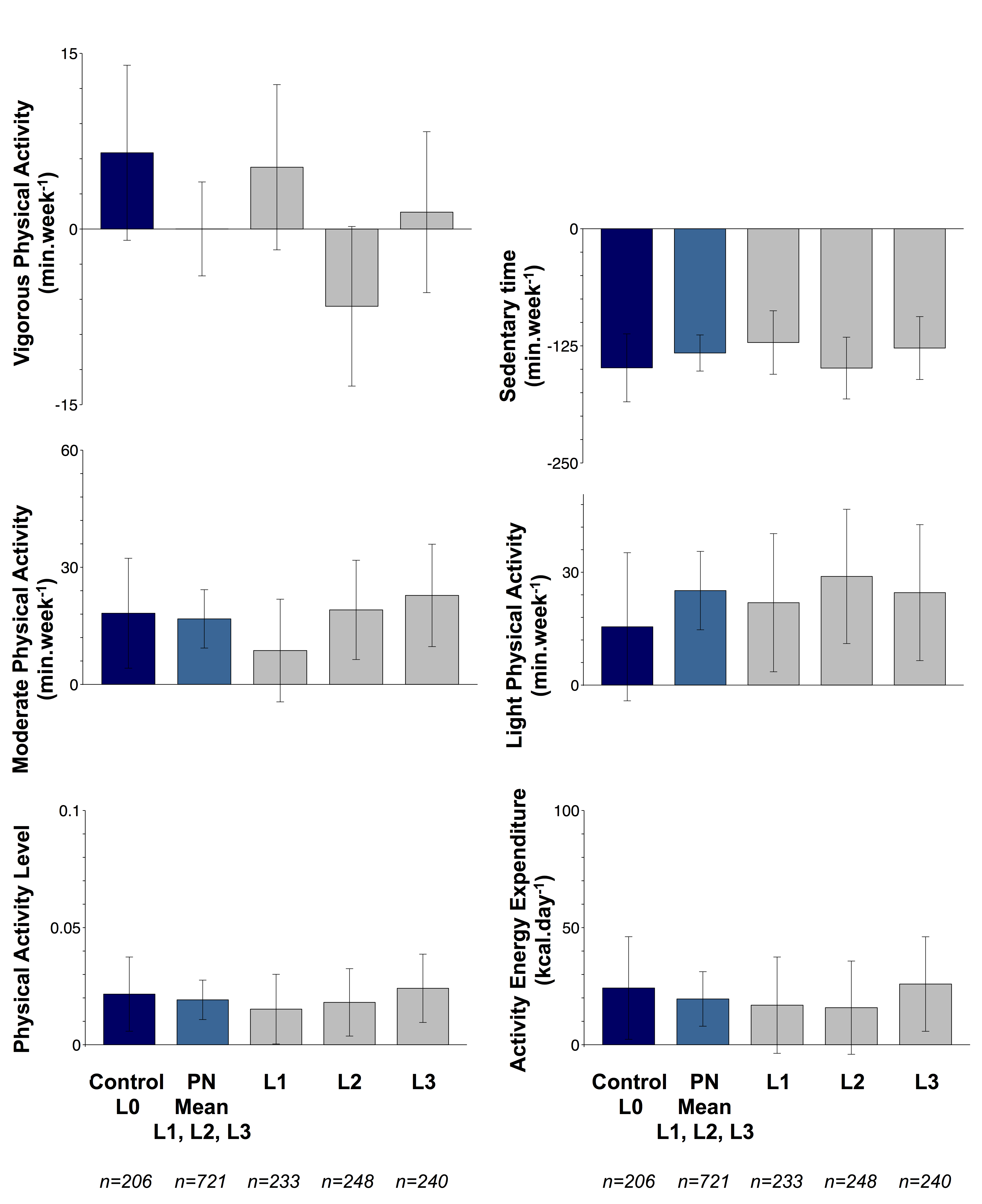

Supplement: Multimedia Appendix 4 [file jmir_v17i10e231_app4.png]

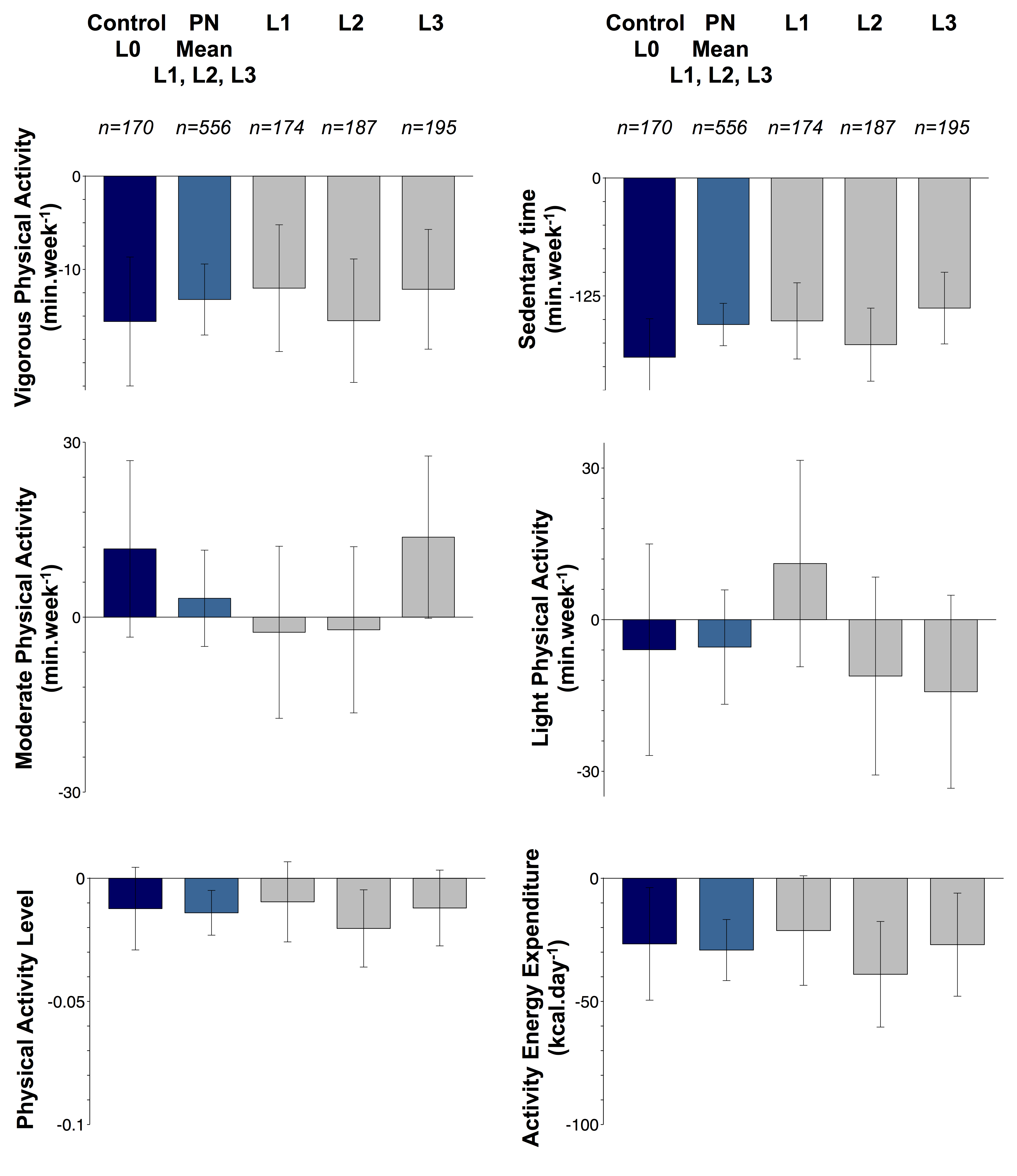

Supplement: Multimedia Appendix 5 [file jmir_v17i10e231_app5.png]

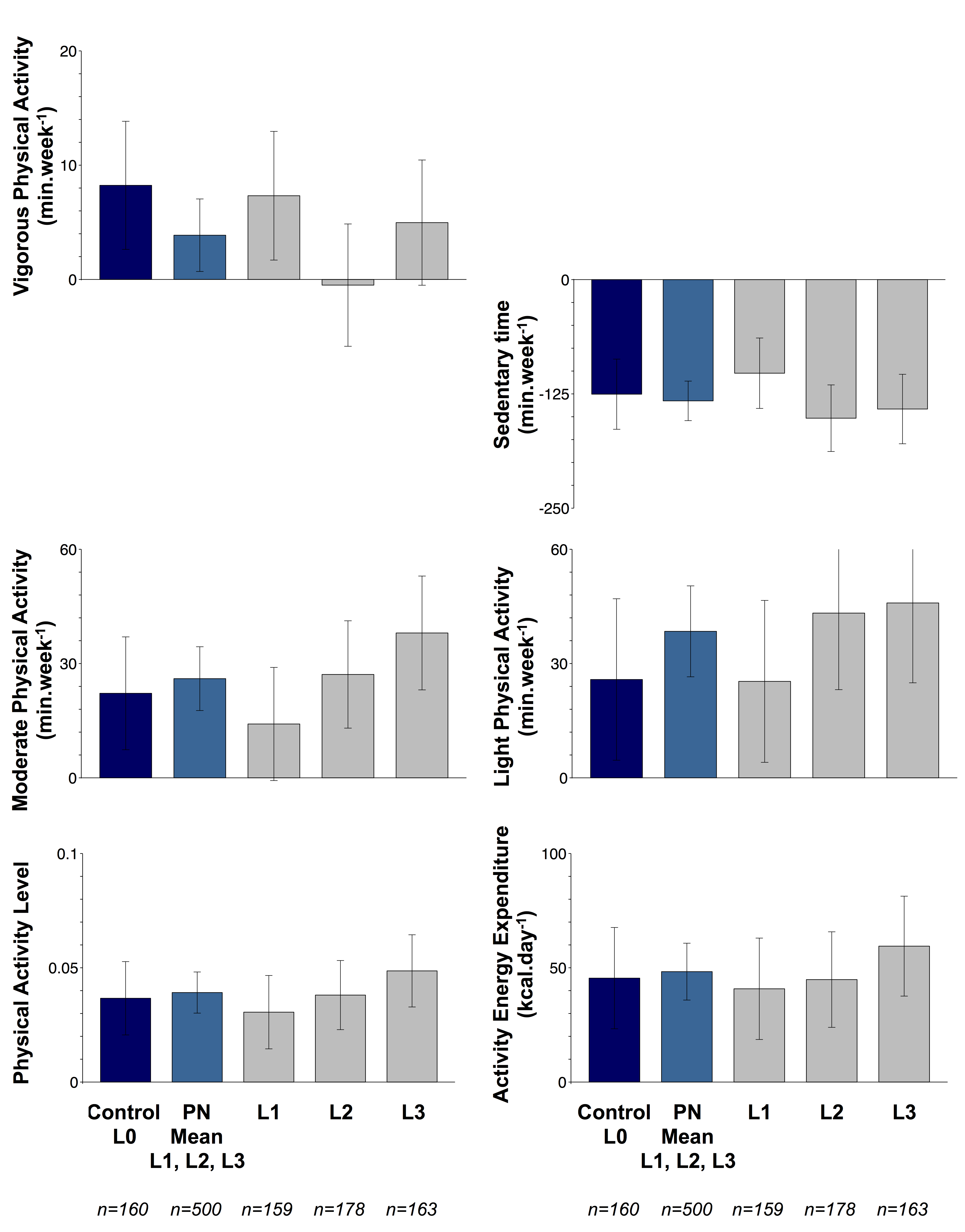

Supplement: Multimedia Appendix 6 [file jmir_v17i10e231_app6.png]

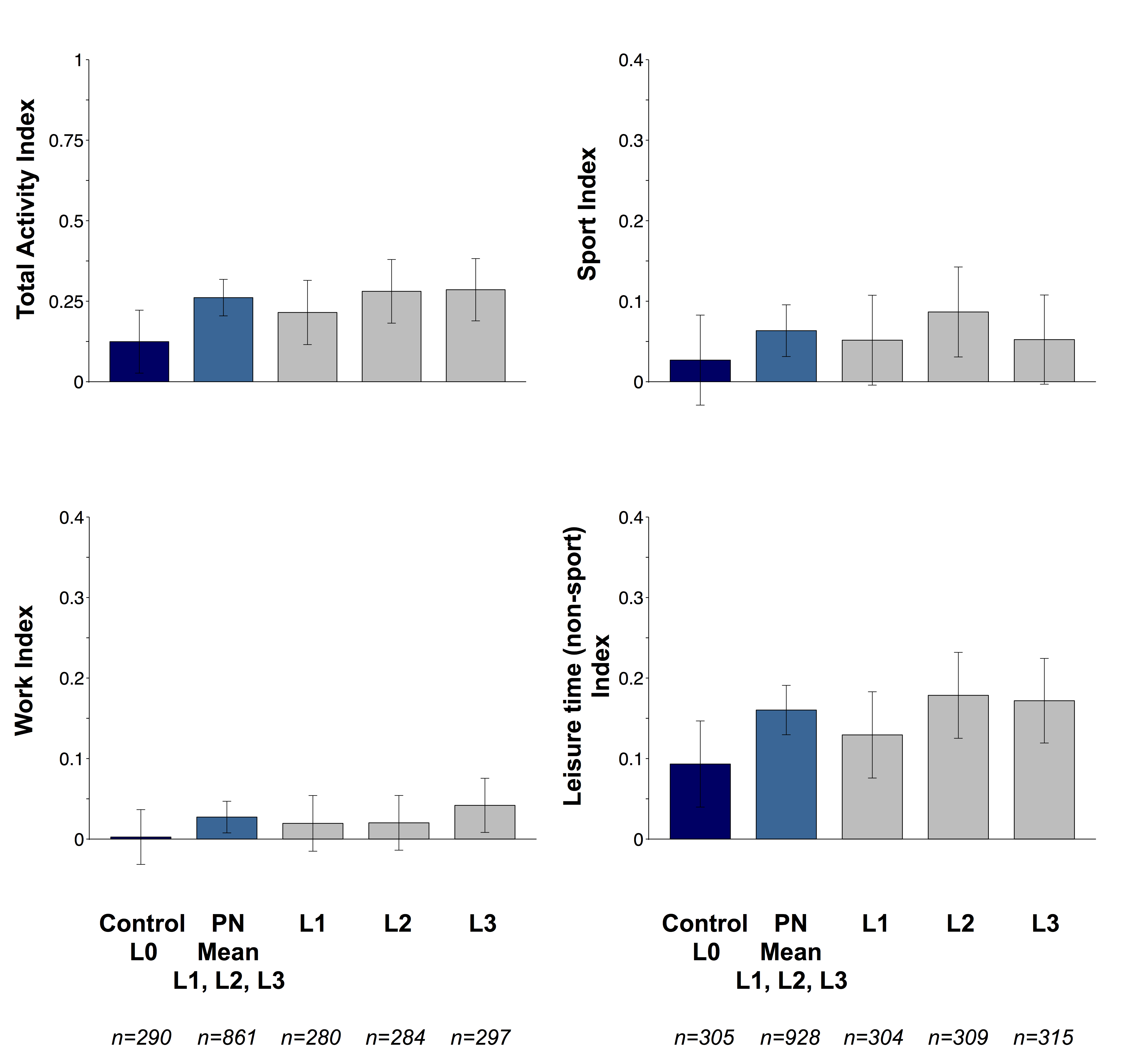

Supplement: Multimedia Appendix 8 [file jmir_v17i10e231_app8.png]

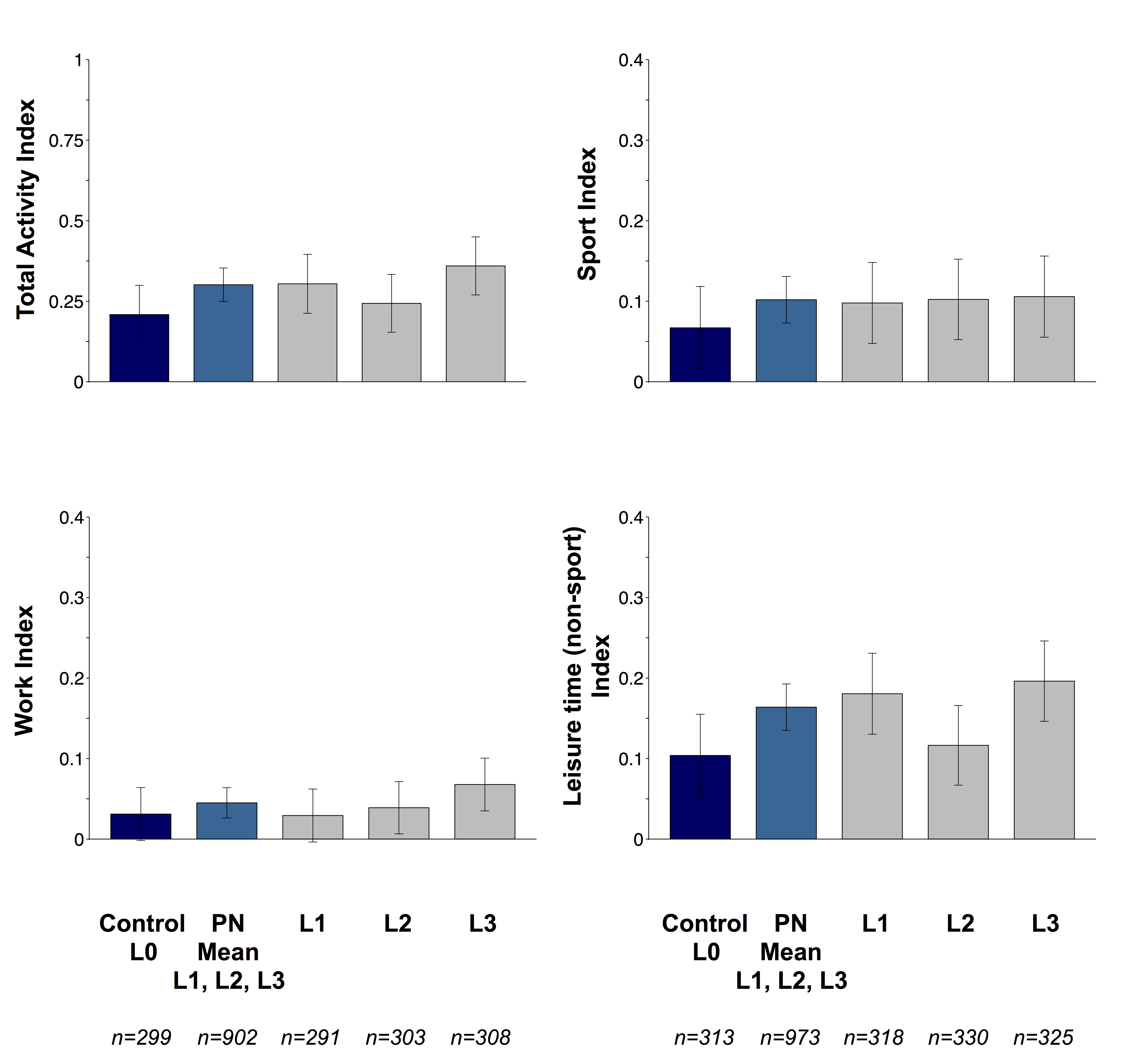

Supplement: Multimedia Appendix 9 [file jmir_v17i10e231_app9.png]

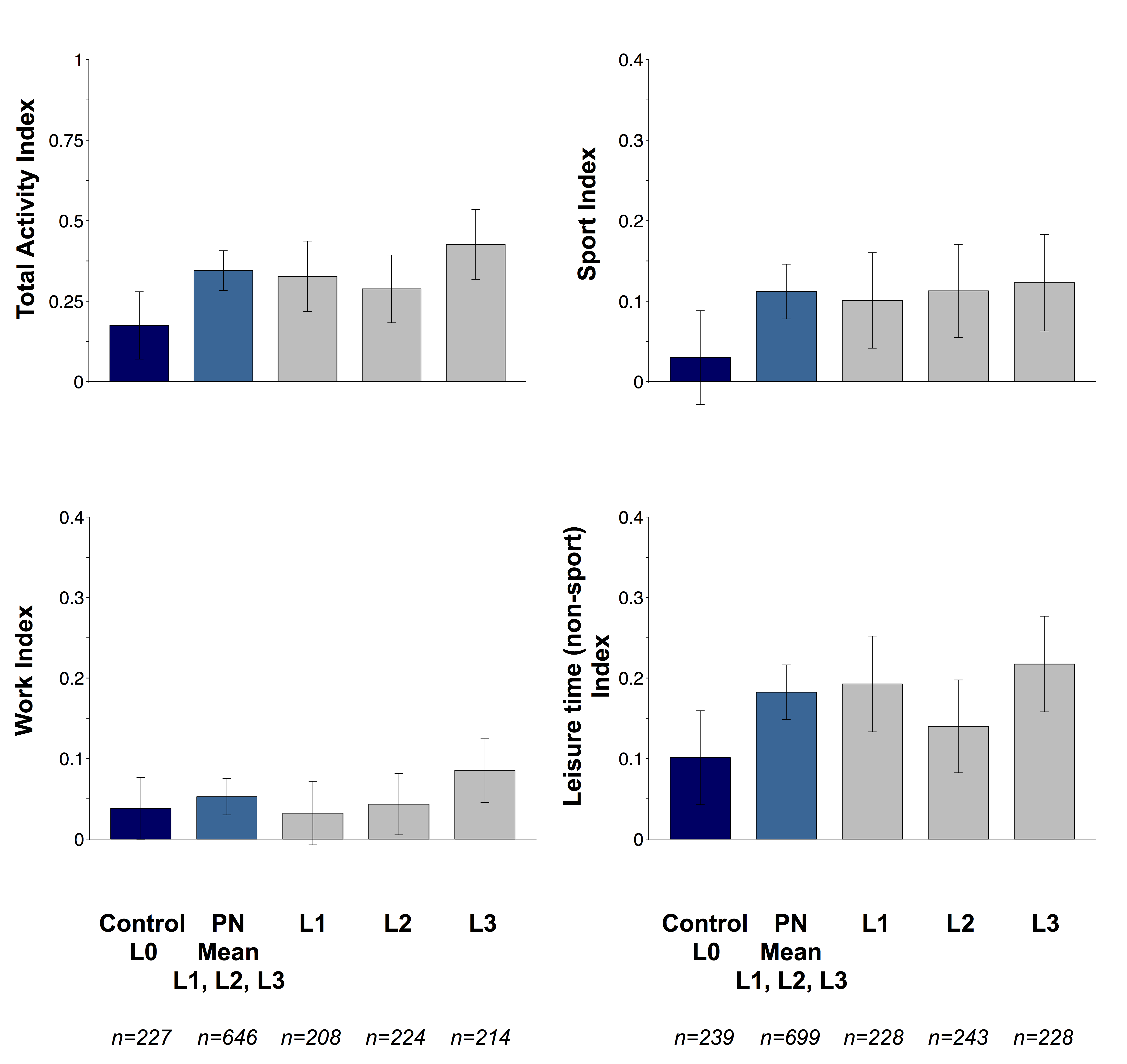

Supplement: Multimedia Appendix 10 [file jmir_v17i10e231_app10.png]
