# Supplementary material for: Effects of a Web-Based Personalized Intervention on Physical Activity in European Adults: A Randomized Controlled Trial
Source: J Med Internet Res. 2015 Oct 14;17(10):e231. doi: 10.2196/jmir.4660 (PMC4642412; doi:10.2196/jmir.4660)
Supplement: Multimedia Appendix 7 [file jmir_v17i10e231_app7.pdf]

**PA components**	**Personalized advice**			***P***	
	Level 1	Level 2	Level 3	L1	L2
	(n=114)	(n=134)	(=131)	vs	vs
				L2+L3	L3
**Objective PA**					
PAL	1.68 (0.1)	1.67 (0.1)	1.67 (0.1)	0.33	0.75
AEE ($kcal.d^{-1}$)	790 (134)	770 (137)	775 (136)	0.26	0.79
Sedentary time ($min.wk^{-1}$)	5204 (248)	5187 (249)	5215 (248)	0.91	0.37
LPA ($min.wk^{-1}$)	503 (131)	480 (132)	476 (130)	0.10	0.79
MPA ($min.wk^{-1}$)	203 (97)	196 (98)	221 (98)	0.66	**0.04**
VPA ($min.wk^{-1}$)	48 (33)	42 (35)	44 (34)	0.20	0.61
Moderate-equivalent PA ($min.wk^{-1}$)	307 (152)	297 (157)	323 (154)	0.88	0.18
Moderate-equivalent PA in bouts ($min.wk^{-1}$)	134 (101)	123 (104)	136 (102)	0.68	0.32
**Self-reported PA**					
Total activity index[a]	7.62 (0.87)	7.84 (0.87)	7.83 (0.87)	**0.006**	0.91
Work index[a]	2.24 (0.3)	2.27 (0.3)	2.29 (0.3)	0.13	0.39
Sport index[b]	2.62 (0.5)	2.69 (0.51)	2.66 (0.51)	0.18	0.48
Leisure time (non-sport) index[b]	2.79 (0.48)	2.9 (0.48)	2.9 (0.48)	**0.008**	0.99

PA, physical activity; L0, level 0; L1, level 1; L2, level 2; L3, level 3; PAL, physical activity level, AEE, activity energy expenditure; LPA, light PA; MPA, moderate PA; VPA, vigorous PA; Moderate-equivalent PA is (MPA + 2 × VPA).

[a]n=220 (L0), 198 (L1), 210 (L2), 207 (L3), 615 (pooled L1, L2, L3). For retired or unemployed individuals, work index, and therefore total index, cannot be calculated.

[b]n=232 (L0), 217 (L1), 230 (L2), 223 (L3), 670 (pooled L1, L2, L3).

Analysis is restricted to participants randomized to Levels 1–3 (L1, L2, L3) receiving personalized advice to increase PA. Data are presented as adjusted means with the corresponding 95% confidence interval. All analyses were adjusted for baseline values, sex, age, country, smoking, baseline BMI, baseline season and change in body weight. In addition for objective PA variables, analyses were adjusted for change in accelerometer wear time.
